# Reprogramming the proteome for cell-free protein expression

**DOI:** 10.1093/synbio/ysaa013

**Published:** 2020-08-10

**Authors:** Tetsuhiro Harimoto

**Affiliations:** Department of Biomedical Engineering, Columbia University, New York, NY, USA

Living cells produce countless numbers of valuable compounds. By engineering these living ‘biofactories’, synthetic biologists have been making novel molecules that can be used for medicine, food, energy and everyday applications. However, crosstalk between engineered modules and host factors can significantly interfere with biomolecule production by competing for common resources. To address this challenge, scientists have been trying to minimize unwanted crosstalk between the host and synthetic networks by deleting proteins that drain resources such as proteases and metabolizing enzymes.

In a recent study in the journal *Nature Communications*, the research team from Cheemeng Tan’s group at the University of California in Davis took a novel approach (1). Instead of minimizing crosstalk, they intentionally re-engineered crosstalk between the host and synthetic networks in order to create a more favorable environment for protein synthesis. This ‘holistic’ engineering approach achieved a global proteome reprogramming and enabled the production of complex proteins.

The research team, led by Luis E. Contreras-Llano and Conary Meyer, utilized a cell-free protein synthesis system to construct consortia of bacteria, each expressing core proteins involved with protein translation. Because expressing multiple proteins in a single strain results in high metabolic burdens to the cells, distribution of the labor between the members of the consortium can improve overall protein expression. In addition, the use of this consortium enabled a rapid investigation of multiple pathways by inoculating different combinations of bacterial cells (2). To prepare the cell-free expression system, the researchers simply obtained cell lysates from the consortia without the need to purify and supplement individual proteins.

The researchers first tested the protein expression capability of their consortia by measuring deGFP levels. They tested 18- and 7-strain consortia expressing various initiation, elongation and termination factors, as well as aminoacyl-tRNA transferases, and found their expression levels to be comparable. In comparison to the wildtype bacteria and commercially available expression system (S30 T7 system), the consortium demonstrated >2-fold increase in deGFP production. Interestingly, when the team investigated the underlying mechanism of the improvement, they found that simple addition of translation machineries did not fully explain the increase in protein synthesis. Thus, they hypothesized that the overexpression of translation machineries in cells led to host reprogramming of the proteome that favors protein synthesis.

To investigate the shift in the proteome, the researchers analyzed protein composition using mass spectrometry. They found that the consortium indeed exhibited a global proteome shift compared to the controls, resulting in changes in the expression level of more than 700 proteins. Importantly, these changes were associated with upregulation in macromolecule synthesis and downregulation of competing factors, such as enzymes involved in glycolysis.

After demonstrating proof-of-concept with deGFP, the team used their novel system to express more complex proteins. They successfully produced functional ferritin and Cas9 proteins, both of which are difficult to synthesize due to their large size and complexity. In addition, utilizing the plug-and-play feature of the consortium, the team added a nuclease inhibitor, Gam protein, to the Cas9 production system, rapidly improving Cas9 expression. Together, these applications demonstrate that the consortium approach works for expressing complex proteins and improving expression quickly and iteratively.

This study highlights the power of taking a more holistic approach to synthetic biology. Traditionally, synthetic biology had focused on the engineering of individual parts in local networks, and rarely investigated their influence in host networks as a whole. In this study, instead of focusing on orthogonality of the synthetic modules, the researchers devised to have local and host networks influence each other to create beneficial environments for specific applications. While the detailed maps of individual networks are still hard to decipher, this holistic framework can offer novel ways to program cells to maximize their functional output.
